# On Measurement of Avoidable and Unavoidable Cost of Alcohol: An Application of Method for Estimating Costs Due To Prior Consumption

**DOI:** 10.3390/ijerph7072881

**Published:** 2010-07-16

**Authors:** Johan Jarl, Ulf-G Gerdtham, Anne Ludbrook, Dennis Petrie

**Affiliations:** 1 Health Economics & Management, Institute of Economic Research, Lund University, P.O. Box 117, SE-221 00 Lund, Sweden; E-Mail: Ulf.Gerdtham@nek.lu.se (U-G.G.);; 2 Center for Primary Health Care Research, Malmö University Hospital, Lund University/Region Skåne, SE-205 02 Malmö, Sweden; 3 Economics Department, Lund University, P.O. Box 117, SE-221 00 Lund, Sweden; 4 Health Economics Research Unit, University of Aberdeen, Polwarth Building, Foresterhill, Aberdeen AB25 2ZD, Scotland, UK; E-Mail: a.ludbrook@abdn.ac.uk (A.L.); 5 Economics Studies, University of Dundee, Perth Rd, Dundee DD1 4HN, Scotland, UK; E-Mail: d.j.petrie@dundee.ac.uk (D.P.)

**Keywords:** alcohol consumption, avoidable costs, lag structure, liver cirrhosis

## Abstract

This study estimates the avoidable and unavoidable costs of alcohol-related, liver cirrhosis inpatient care, controlling for the lag structure and period of decline in disease risk. Lag structures with different lengths are applied to the exposure to risk from alcohol consumption, which allows for differentiation between avoidable and unavoidable cases due to prior consumption. A lag length of 20 (men) and 23 (women) years (expected remaining life years) gives a total cost of 592 million SEK. Given alcohol consumption is reduced to zero, 72% of cost could potentially be avoided. It is important to account for the length and structure of the risk decline following a consumption change as this substantially affects the estimates.

## 1. Introduction

A number of studies have estimated the total cost of alcohol (COA) consumption [1–3]. Although the results of these studies are interesting, their relevance for policy is limited as they neither assist with resource allocation decisions nor accurately specify areas that should be targeted by interventions. The main advantage of COA studies is rather to serve as a starting point for further studies, by defining and valuing the current costs and benefits connected to current and previous alcohol consumption [4]. Economic evaluations, however, are needed to choose between potential alcohol interventions, and this requires detailed knowledge about the potential future benefits from reductions in alcohol consumption. Thus, one problem with COA studies is that they do not differentiate between costs which can be avoided and those which are unavoidable at a particular point in time. For that reason, avoidable costs, costs that can be avoided given a reduction in current and future risky alcohol use, are of primary interest as it is through this concept that more accurate prioritisation and ranking between policies/interventions may be based.

As compared with traditional COA studies, the counterfactual scenario needs to be changed when estimating the avoidable cost. COA studies generally use a counterfactual scenario of a society without the adverse effects of current or previous alcohol consumption. A more realistic scenario is to compare the expected future cost of alcohol consumption, with a society where alcohol consumption is reduced at a certain point in time. This may result from a hypothetical or existing intervention with known effect. Following the definition in international guidelines, the total cost of alcohol is made up of two parts, the avoidable and unavoidable cost [4]. The unavoidable part consists of already existing alcohol-related diseases and new cases due to prior consumption, plus cases due to continued (irreducible) consumption (consumption which cannot be reduced further regardless of the degree and number of interventions implemented, given current level of knowledge). The avoidable part is thus those cases that are amenable by interventions and behaviour changes [4], which can be difficult to define for a particular population, at a given point in time, given the large range of interventions or possible combinations. We point out that it is also important to identify the future cost due to prior consumption separately from continued irreducible consumption as this will give an indication with regards to the potential future cost saving of developing new, more effective, interventions. The point being that what is continued irreducible consumption today not necessarily remains so tomorrow. Thus, the current study makes this distinction. In order to make the difference of the current definition and the definition in the international guidelines clear, we refer to the current as unavoidable cost due to prior consumption.

The estimation of avoidable costs is normally justified by its contribution to: (1) prioritising expenditure of public funds; (2) facilitating analysis of intervention programs as well as the design of such programs; (3) identifying information gaps both in research and data collection; and (4) improving the analysis of the cost-effectiveness of policies and interventions [4]. A more general and maybe more important justification is that estimation of avoidable costs would probably foster research and development in areas where potential cost savings are significant [5]. This latter general justification point out the importance of what was discussed above, that unavoidable cost due to prior consumption is differentiated from unavoidable cost due to continued irreducible consumption.

Estimating the avoidable cost of alcohol consumption is a new area of research. Following the publication of guidelines for estimating the social cost of substance abuse by the World Health Organisation (WHO) in 2003 [6], Health Canada initiated a workshop in 2005 with the aim of producing theoretical guidelines for estimating the avoidable cost of substance abuse. As well as working towards the development of sound methodologies and approaches, the intention was to encourage and provide guidance for pilot studies in this area. Although the resulting guidelines build on the most recent knowledge in all relevant areas such as epidemiology and criminology, it is expected that empirical work will encounter a multitude of challenges which will develop the guidelines [4]. To date, three robust attempts have been made to estimate the avoidable costs of alcohol consumption [7–9], two of which are a direct result of the theoretical guidelines using the same definitions [7,8].

A Canadian study estimated the avoidable health care, crime and indirect costs following six potential interventions in the alcohol field while Collins and Lapsley estimate the avoidable alcohol-related cost following seven potential interventions for Australia [7,8]. Although both studies use some potential interventions to help determine the irreducible consumption and thus avoidable cost, other approaches to define the irreducible consumption are also tested. All effects from reduction in alcohol consumption are considered immediate in both studies, *i.e.*, no time characteristics of risk decline were accounted for in the estimations, due to data limitations. This is dealt with to some extent in a study from the UK, which investigates the effect on alcohol-related harm from different alcohol-related interventions [9]. The authors assume a lag length of 10 years before the full health effect of a consumption change is manifested. It is further assumed that the lag structure is linear, *i.e.*, that each year has an equal proportion of risk decline. In general, the report includes harm related to health, crime and employment and a total of 53 different scenarios were estimated showing avoidable effects and costs [9].

Thus, a major obstacle to allowing for temporal characteristics in prior research has been a lack of data, forcing the issue to be overlooked or handled with somewhat subjective assumptions. The current study develops a method for handling the time characteristic of risk decline following a consumption change based on population information available for liver cirrhosis. In order to illustrate the method in the current study we also estimate the unavoidable cost due to prior consumption.

### 

#### Aim

The aim is to extend and apply methods to estimate the societal avoidable and unavoidable cost of inpatient care due to liver cirrhosis, accounting for the time characteristics of risk decline. We use a counterfactual scenario where alcohol vanishes overnight, *i.e.*, where effects of prior consumption still lingers but where present and future consumption is abolished, in order to estimate the unavoidable cost due to prior consumption as discussed above. The results for men and women are presented separately, but the calculations for women are shown in more detail in the text while the detailed results for men are found in the appendix. The focus is on liver cirrhosis as it has been shown to have a very strong link to alcohol consumption and thus alcohol-related harm [10]. A crucial assumption is that the age and gender characteristics of the population and the distribution of alcohol consumption in the actual scenario are constant, *i.e.*, children who grow up start consuming according to the distribution in year zero. In the counterfactual scenario, children who grow up do not start consuming alcohol. Alternatively, the population at year zero could be fixed and followed until death. However, the results of such a study would not be comparable with the COA literature. The base of this study is the recent Swedish COA study [11,12] and this should be seen as a step towards extending methods to allow for the future estimation of the avoidable cost.

The Swedish COA study estimated a total net cost of alcohol consumption in 2002 of 20.3 billion Swedish kronor (SEK). The cost of inpatient liver cirrhosis care was estimated to be 37 million (SEK) out of which women’s consumption stood for 12 million (SEK). A total of 328 (677) liver cirrhosis cases (ICD-10 codes K70 & K74) were attributed to alcohol consumption for women (men) with a unit cost of 36,796.75 SEK. That is, in the counterfactual scenario of no adverse effects of alcohol consumption 328 cases would have been averted for women, as shown in Table 1 [12].

## 2. Method

The total cost estimated in the Swedish COA study is a yearly moment-in-time assessment of the cost. However, the increased risk of disease is expected to decline gradually over time following a reduction of alcohol consumption, *i.e.*, the temporal path of decline in disease risk needs to be accounted for when estimating the avoidable cost. This is challenging, especially as there is a considerable lack of research on the time path of disease risks. To the authors’ knowledge, no individual level study exists that investigates how the risk of liver cirrhosis changes over time following a change in alcohol consumption.

However, the lag structure of population consumption on liver cirrhosis mortality has been investigated somewhat in aggregated time series analyses [13]. It is generally found that the lag structure is surprisingly short and that changes in alcohol consumption have an immediate, substantial effect on mortality rates [13–15]. Additional lags affect the mortality rates at a decreasing rate, which is surprising as alcoholic liver cirrhosis takes several years to develop [13].

### 2.1. Lag Structure

This study employs the lag structure modelled by Norström [13] which includes both the short- and long-term effects of changes in alcohol consumption, originally suggested by Skog [16]:

(1) $$w_{i}=p\lambda_{1}^{i}\;+\;(1-p)\;\lambda_{2}^{i}$$

where *w_i_* is the weight of consumption in year *i*, *λ_1_* and *λ_2_* are the geometrically declining lag parameters for the short- and the long-term effects respectively and *p* is the relative importance of the short term effect [13]. In the same study, using Swedish data, Norström concludes that appropriate parameter values are 0.50 for *λ_1_*, 0.93 for *λ_2_*, and 0.80 for *p*, which were close to the values obtained in an earlier study using British and French data [16]. The short-term effect is thus considered to be stronger than the long-term effect, although it dies out faster. It follows that the lag weights decline at a decreasing rate, not reaching zero within a human lifetime. However, the weight quickly becomes small (below 0.1 after 9 years, below 0.05 after 19 years, and below 0.01 after 41 years) making the inclusion of an additional lag of diminishing importance, especially considering the need to discount future costs. A 3% discount rate will be used in the current study, following the Swedish COA study [11].

The UK study assumes a time period of 10 years (including the initial year, *i.e.*, the immediate effect) between consumption change and the full effect [9]. The assumption is based on the time period for developing a disease, averaged over all alcohol-related chronic diseases. A 10 year lag period has also been used by Ramstedt when estimating the effect of changes in per capita consumption on liver cirrhosis mortality [17]. Following these prior studies, the current paper also uses a 10 year lag period, in addition to an “average expected remaining life years” period. This will establish the sensitivity of the results with regard to the length of the period for disease risk decline. The appropriate length of the lag period will be dependent on the lag structure and the chosen discount rate and is therefore expected to be disease specific. The weights for the 10 year lag period are shown in Table 2.

The weights are summed (=3.073) and a proportion for each year is calculated based on the ten year time period. That is, 33% of the total reduction in liver cirrhosis following abstention occurs immediately (1/3.073) while 3% occurs in the tenth year (0.11/3.073). After ten years the full effect of the risk decline are manifested, following the assumption above.

As the lag structure is based on aggregate consumption, it is not known to what extent these weights are different between consumption groups. With such data, it would have been possible to have different weights and even different time periods depending on level of consumption. Without such data, an additional assumption is required stating that the risk decline following consumption change is proportionally the same between individuals, independent of level of original consumption. Regarding liver cirrhosis, this assumption is of less concern as the disease normally is considered to develop from long-term consumption [13] and increased risk of disease is based on level of consumption [17 and referred literature] and not from periodic binge drinking.

### 2.2. Exposure Based Lag Structure

The lag structure should be modelled directly on the relative risk of disease as this will allow the estimation of new cases over the time period. The results of the alternative approach, *i.e.*, modelling the temporal characteristics on the outcomes, should be interpreted as the distribution of the length of treatment, *i.e.*, a focus on palliative and curative services [5]. That is, the study needs to be exposure based rather than outcome based [5]. The advantage of focusing on exposure is that it allows for differentiation between avoided and delayed effects, which is theoretically more appropriate for avoidable cost calculations. The relative risk (RR) is simply:

(2) $$\frac{P_{\text{exp}\text{osed}}}{P_{\text{un}\text{exp}\text{osed}}}\;=\;\text{RR}$$

where *P* is the probability of an event for exposed and unexposed respectively. The probability of an event for those exposed comprises two components, the probability of an event for those unexposed (baseline) and the change in risk following exposure:

(3) $$P_{\text{un}\text{exp}\text{osed}}\;+\;\Delta P\;=\;P_{\text{exp}\text{osed}}$$

By inserting (3) in (2) and rearranging, we get:

(4) $$(\text{RP}*\;P_{\text{un}\text{exp}\text{osed}})\;-\;P_{\text{un}\text{exp}\text{osed}}\;=\;\Delta P$$

If we now assume a change in the probability of an event for those exposed, which will affect the relative risk, this can be modelled as:

(5) $$\frac{P_{\text{un}\text{exp}\text{osed}}\;+\;(1+x)\;\Delta P}{P_{\text{un}\text{exp}\text{osed}}}\;=\;{\text{RR}}_{\text{new}}$$

By substituting (4) into (5) and simplifying, the following is given:

(6) $$\text{RR}(1+x)\;-\;x\;=\;{\text{RR}}_{\text{new}}$$

The last formula thus gives the new relative risk based on a change in the probability of an event in the exposed group when only the original relative risk and the actual change in probability is known. Applying equation (6), where *x* is the negative cumulative proportion of weights in Table 2, in the case of liver cirrhosis gives the relative risks after abstention (Table 3).

It is now possible to calculate the number of new cases following abstention. The following formula is used for the calculation of the alcohol attributable fraction (AAF):

(7) $$\text{AAF}\;=\;\frac{\sum_{i=1}P_{i}*({\text{RR}}_{i}-1)}{1+\sum_{i=0}P_{i}*({\text{RR}}_{i}-1)}$$

where *i* denotes the drinking categories (see Table 3), P is the prevalence rate and RR is the relative risk of the *i*:th category. As the distribution of alcohol consumption differs over age, different AAFs are calculated for seven different age groups (Table 1). Given the fact that the number of alcohol related cases are reduced each year in the counterfactual scenario, it follows that the total number of liver cirrhosis cases (alcohol and non-alcohol related cases) are also reduced each year. That is, the number of cases to which the AAF is applied is adjusted for each consecutive year according to the number of alcohol-related cases avoided in the year before. For example, there are originally 902 female cases of liver cirrhosis in Sweden in 2002 (Table 1), following abstention 74 alcohol-related cases (out of 328) are avoided in year 0 leaving 828 cases in the beginning of year 1.

## 3. Results

In Table 4 are the estimated cases and costs of liver cirrhosis for women with a 10 year long lag period shown. The inpatient health care costs for alcohol attributable liver cirrhosis comprises two parts, one unavoidable part due to prior alcohol consumption, and one avoidable part that would disappear if alcohol vanished overnight in year 0. Over the full ten year period, the unavoidable part sums to 30 million SEK while the avoidable part sums to 76 million SEK, giving a total cost of alcohol-related liver cirrhosis due to current consumption pattern of 106 million SEK, *i.e.*, potentially 72% of the total cost of alcohol-related liver cirrhosis is avoidable over a 10 year period for women. In the Swedish COA study, the cost for year 0 was estimated to be 12 million SEK. Table 4 shows that this cost is 23% avoidable, a proportion that is increased for each consecutive year, culminating at 100% avoidable by the tenth year. For each additional year, starting with year 11, 328 cases are avoidable in the counterfactual scenario, although the costs are more heavily discounted. This raises the question of when to stop counting. In the current situation, it makes some sense to stop after 10 years as, given the assumptions, the theoretical minimum risk is reached (the lowest possible risk). However, if the 10 year period assumption is relaxed, the theoretical minimum risk will be reached at a different time point. Table 5 shows the number of avoidable and unavoidable cases and costs for both women and men. The avoidable proportion is slightly lower for men (71%).

As noted above, Meier *et al.* is the only existing study that controls for the time characteristic of risk decline. This is done by assuming a 10 year lag period with a linear risk decline [9]. To allow for comparison, the results from Table 5 are reproduced below (Table 6) using the same assumption. It is shown that failure to account for the stronger short-term effect, compared to the long-term effect, will underestimate the amount of cost that could be avoided. It is even the case that the avoidable cost is lower than the unavoidable cost, due to the fact that avoidable costs are more heavily discounted as this is predominantly a late effect.

### 3.1. Lifelong Lag

The 10 year lag period applied above was used based on assumptions in earlier studies. However, the lag structure attained from Norström [13] implies that the increased risk of developing liver cirrhosis due to alcohol consumption compared to abstention never really disappears. Thus, alcohol consumers can expect to have an increased risk of disease until death, even if they become abstainers. This section will therefore calculate the increased risk and associated costs for the expected remaining life years. On average liver cirrhosis patients in Swedish inpatient care were 60 years of age (the same for women and men) in 2002. The corresponding expected remaining life years for these patients were 24 and 21 years for women and men respectively [19].

The associated costs are, as expected, higher when the lag period is increased, given that counting stops when the theoretical minimum is reached. The total cost for the lifelong lag sums to 211 million SEK, see Table 7, *i.e.*, almost twice the cost of the 10 year lag. However, the proportion of the costs that theoretically could be avoided by complete societal abstention is only marginally affected, increasing by 1 percentage point to 73%. This is to some extent a result of discounting, putting less weight on later years. The corresponding cost for men is 381 million SEK out of which 72% are avoidable over the next 20 years, see Table 8.

### 3.2. Sensitivity Analysis

One sensitivity analysis was conducted regarding the parameter values in equation 1. Instead of the values estimated by Norström [13] the values estimated from British and French data by Skog [16] were used (0.60 for λ_1_, 0.95 for λ_2_, and 0.85 for p). Thus, the short term effect is stronger and declines less rapidly. However, this is countered by an increase in the long-term effect. The results show that using the values from the Skog study would increase the number of cases and costs that are unavoidable due to prior consumption, although marginally, see Table 9.

## 4. Discussion

Even if alcohol consumption magically disappeared overnight, Swedish society would still suffer the cost of alcohol-related liver cirrhosis in the magnitude of 165 million SEK, based on the remaining life years lag structure. However, a cost in the magnitude of 428 million would have been avoided, showing the maximum theoretical gain from alcohol interventions with regard to liver cirrhosis.

Prior studies have used a 10 year long lag structure which, as expected, results in substantially lower costs. However, in order for the estimates to be comparable, the cost in the 10 year lag period should be extrapolated over expected remaining life years where the full effect of the risk decline is added to each additional year. As noted above, the lag structure obtained declines at a decreasing rate. It thus appears that the most appropriate length is to use the expected remaining life years as the increased risk due to alcohol consumption does not reach zero within a normal human life span. It should be noted that this is in regard to how long the unavoidable cost due to prior consumption needs to be considered but not how long the benefits from reducing alcohol consumption should be considered as the beneficial effects will continue after the adverse effects of prior consumption has disappeared.

The Swedish COA study estimated that the cost of alcohol-related liver cirrhosis for inpatient care in 2002 amounted to 37 million SEK. This is the cost that would be avoided in one year if the full effect of a consumption change occurred immediately. Although the current study shows that the immediate effect is substantial, assuming that the full sum could be saved in 2002 would be vastly misleading. The immediate cost saving would only be 17% of the total cost, as estimated in the current study.

The lag structure employed in the current paper indicates a higher relative importance of the short-term effect. The proportionally higher avoidable, compared with unavoidable, cost is largely driven by this. The comparison of this study’s result with a linear risk decline, as used in prior research [9], shows that the difference is substantial. It is expected that failure to account for the strong short term effect could bias cost-effectiveness ratios in future economic evaluations.

One limitation in the Canadian and Australian avoidable cost studies, which is also pointed out by the respective authors, is that the lag structure cannot be estimated due to data limitations. The full effect of risk decline following a change in consumption could thus mistakenly be interpreted to be manifested immediately, *i.e.*, in year zero [7,8]. This problem is discussed in the reports, and the Canadian study even goes so far as to conduct a literature review regarding the current knowledge of the lag structure for different alcohol-related diseases [7]. In the case of the current hypothetical intervention, this interpretation of an immediate effect would make the estimate of avoidable cost in year zero equal to the total cost of alcohol consumption, which certainly is not the case. The UK study takes something of a middle ground, estimating the avoidable cost of existing interventions although applying a method that makes it easy to differentiate between avoidable and total cost even if the current hypothetical intervention was used [9].

The use of economic evaluations is much less developed in the alcohol field compared with many other areas of health economics. This is due to the fact that the impact of alcohol is complex and multi-dimensional. It affects for example crime, productivity, health and quality of life which make evaluations difficult to carry out. The result of this is a limited knowledge about the cost-effectiveness of interventions and policies targeting alcohol-related harm. While estimating the theoretically avoidable cost of alcohol consumption is of limited importance on its own, using this information in connection with economic evaluations of interventions will provide important direction for policy-makers and society. Estimating the theoretically avoidable cost will facilitate future economic evaluations. It is thus important that the current study is considered in connection with future economic evaluations in the alcohol field, thereby increasing the practical importance of research on the cost of alcohol.

One limitation of the current study is that it has not been possible to differentiate between new and existing cases in year 2002, *i.e.*, the cases developed prior to the hypothetical disappearance of alcohol consumption need to be identified in order to give a complete picture of the avoidable and unavoidable cost, due to prior consumption. Liver cirrhosis patients who stop consuming alcohol are treated for about 3–5 years (shorter for those who continue to drink). However, it is not currently clear whether they are registered as inpatient cases beyond the initial hospitalisation spell. The incidence-prevalence ratio at year zero could not be studied at this time due to data limitations but should be investigated in future research. Such a study, however, will have to deal with the difficulty of not only separating existing from new cases in year zero but also controlling for those patients who do not stop consuming alcohol.

Another issue that needs to be accounted for in future studies of the avoidable cost is the trend of alcohol consumption. If per capita consumption is rising over time after year zero, the counterfactual scenario will result in even larger avoidable cost, and vice versa, for a decline in consumption.

Another type of study that is needed more desperately than any other, in order to estimate the avoidable cost of alcohol, is investigations of the disease-specific risk decline following reduction in consumption for all alcohol-related diseases. Currently this information is largely lacking. Unless this data could be attained through epidemiological studies, it could potentially be studied using the approach in Norström [13] and Skog [16] on which the current study relies. Future developments in this area should also considerer the potentially different effects on risk decline following different length, volume and pattern of consumption. It is possible that the effect of drinking cessation on risk decline is dependent on how long the individual has consumed alcohol and in what manner. Besides, the current study has assumed the same proportional risk decline irrespective of consumption level. This need not be correct as it is possible that the function of risk decline is different between different consumption groups.

## Figures and Tables

**Table 1:** Alcohol attributable liver cirrhosis cases for women, inpatient care.

**Age**	**Cases**	PAF*	**Cases due to alcohol**
0–14	9	0	0
15–17	2	0.46	1
18–29	9	0.52	5
30–49	163	0.47	76
50–64	359	0.44	157
65–79	312	0.26	80
80+	48	0.20	10

Total:	902		328

Columns and calculations may not sum due to rounding.

* Population attributable fraction

**Table 2:** Weights of the lag structure.

**Year**	**Weight**	**Proportion per lag**	**Cumulative proportion**

0	1.00	0.33	0.33
1	0.59	0.19	0.52
2	0.37	0.12	0.64
3	0.26	0.08	0.72
4	0.20	0.06	0.79
5	0.16	0.05	0.84
6	0.14	0.05	0.89
7	0.13	0.04	0.93
8	0.12	0.04	0.97
9	0.11	0.03	1.00

**Table 3:** Relative risk of liver cirrhosis for women following abstention with a 10 year lag period.

Year*	**Abstinence**	**Low**	**Hazardous**	**Harmful**
−1	1.00	1.30	9.50	13.00
0	1.00	1.20	6.73	9.09
1	1.00	1.15	5.11	6.81
2	1.00	1.11	4.08	5.35
3	1.00	1.08	3.36	4.33
4	1.00	1.06	2.81	3.55
5	1.00	1.05	2.35	2.91
6	1.00	1.03	1.96	2.36
7	1.00	1.02	1.61	1.86
8	1.00	1.01	1.29	1.41
9	1.00	1.00	1.00	1.00

* Year−1 is the current situation while year 0 is the immediate effect of abstention in the counterfactual scenario. The same drinking categories are used as in the Global Burden of Disease study [18]. Abstinence (no alcohol within last year), low consumption (women 0–19.99 grams (gr) pure alcohol per day, men 0–39.99), hazardous consumption (women 20–39.99, men 40–59.99), and harmful consumption (women 40+, men 60+)

**Table 4:** Avoidable and unavoidable liver cirrhosis cases and costs for women, 10 years.

**Year**	**Cases**	Cases avoided*	**Unavoidable cost, undisc.**	**Avoidable cost, undisc.**	**Unavoidable cost, disc.**	**Avoidable cost, disc.**
−1	328	0	12,071,582	0	12,071,582	0

0	254	74	9,352,724	2,718,858	9,352,724	2,718,858
1	183	146	6,716,043	5,355,540	6,520,430	5,199,553
2	132	196	4,848,215	7,223,368	4,569,907	6,808,717
3	98	230	3,594,030	8,477,552	3,289,047	7,758,161
4	73	255	2,698,800	9,372,783	2,397,848	8,327,596
5	54	274	1,998,737	10,072,846	1,724,128	8,688,925
6	38	290	1,410,098	10,661,484	1,180,935	8,928,825
7	24	304	892,527	11,179,055	725,706	9,089,595
8	12	316	426,045	11,645,538	336,324	9,193,095
9	0	328	0	12,071,582	0	9,251,863

Total	868	2,413	31,937,218	88,778,606	30,097,050	75,965,189

* Compared to the original situation, *i.e.*, year −1.

**Table 5:** Avoidable and unavoidable liver cirrhosis cases and discounted costs, 10 years.

	**Cases unavoidable**	**Cases avoidable**	**Costs unavoidable**	**Costs avoidable**
Women	868	2,413	30,097,050	75,965,189
Men	1,815	4,951	62,953,983	155,782,017

Total	2,683	7,363	93,051,032	231,747,206

**Table 6:** Avoidable and unavoidable liver cirrhosis cases and discounted costs, 10 years and linear risk decline.

	**Cases unavoidable**	**Cases avoidable**	**Costs unavoidable**	**Costs avoidable**
Women	1,570	1,710	53,479,839	52,582,399
Men	3,267	3,499	111,241,973	107,494,027

Total	4,837	5,209	164,721,812	160,076,426

**Table 7:** Avoidable and unavoidable liver cirrhosis cases and costs for women, expected remaining life years.

**Year**	**Cases**	Cases, avoided*	**Unavoidable, cost, undisc.**	**Avoidable, cost, undisc.**	**Unavoidable, cost, disc.**	**Avoidable cost, disc.**
−1	328	0	12,071,582	0	12,071,582	0

0	272	56	10,025,817	2,045,766	10,025,817	2,045,766
1	219	109	8,043,836	4,027,746	7,809,550	3,910,433
2	179	149	6,601,647	5,469,935	6,222,686	5,155,939
3	152	176	5,604,762	6,466,820	5,129,152	5,918,056
4	133	195	4,884,660	7,186,922	4,339,957	6,385,487
5	118	211	4,323,788	7,747,795	3,729,737	6,683,316
6	105	223	3,856,319	8,215,264	3,229,606	6,880,154
7	94	234	3,448,125	8,623,457	2,803,641	7,011,660
8	84	244	3,081,670	8,989,912	2,432,699	7,096,720
9	75	253	2,747,595	9,323,987	2,105,803	7,146,060
10	66	262	2,440,510	9,631,072	1,815,969	7,166,422
11	59	269	2,156,983	9,914,600	1,558,250	7,162,518
12	51	277	1,894,576	10,177,007	1,328,817	7,137,948
13	45	283	1,651,391	10,420,191	1,124,517	7,095,643
14	39	289	1,425,848	10,645,735	942,653	7,038,085
15	33	295	1,216,566	10,855,016	780,868	6,967,422
16	28	300	1,022,314	11,049,268	637,072	6,885,539
17	23	305	841,973	11,229,609	509,408	6,794,098
18	18	310	674,519	11,397,064	396,209	6,694,574
19	14	314	519,009	11,552,574	295,983	6,588,271
20	10	318	374,575	11,697,008	207,393	6,476,350
21	7	322	240,414	11,831,168	129,234	6,359,836
22	3	325	115,785	11,955,798	60,427	6,239,641
23	0	328	0	12,071,582	0	6,116,571

Total	1,826	6,047	67,192,682	222,525,297	57,615,449	152,956,508

* Compared to the original situation, *i.e.*, year−1.

**Table 8:** Avoidable and unavoidable liver cirrhosis cases and discounted costs, expected remaining life years.

	**Cases, unavoidable**	**Cases, avoidable**	**Costs, unavoidable**	**Costs, avoidable**
Women	1,826	6,047	57,615,449	152,956,508
Men	3,305	10,226	106,894,811	274,601,315

Total	5,131	16,274	164,510,260	427,557,824

**Table 9:** Sensitivity analysis of avoidable and unavoidable liver cirrhosis cases and discounted costs, 10 years for women.

	**Cases, unavoidable**	**Cases, avoidable**	**Costs, unavoidable**	**Costs, avoidable**
Baseline	868	2,413	30,097,050	75,965,189
Skog, 1984	871	2,410	30,267,354	75,794,884

## Appendix

**Table A1.** Alcohol attributable liver cirrhosis cases for men, inpatient care.

**Age**	**Cases**	**PAF***	**Cases due to alcohol**
0–14	1	0.00	0
15–17	0	0.40	0
18–29	4	0.60	2
30–49	235	0.51	120
50–64	814	0.46	371
65–79	468	0.35	162
80+	67	0.32	22

Total	1 589		677

Columns and calculations may not sum due to rounding.

* Population attributable fraction

**Table A2.** Avoidable and unavoidable liver cirrhosis cases and costs for men, 10 years.

**Year**	**Cases**	**Cases avoided***	**Unavoidable cost, undisc.**	**Avoidable cost, undisc.**	**Unavoidable cost, disc.**	**Avoidable cost, disc.**
−1	677	0	24,895,662	0	24,895,662	0

0	532	145	19,574,946	5,320,716	19,574,946	5,320,716
1	384	293	14,126,092	10,769,570	13,714,653	10,455,893
2	276	400	10,168,165	14,727,498	95,84,470	13,882,079
3	204	473	7,502,131	17,393,531	68,65,513	15,917,545
4	153	524	5,613,419	19,282,243	4,987,450	17,132,024

5	113	564	4,148,641	20,747,022	3,578,654	17,896,563
6	79	597	2,923,446	21,972,216	2,448,340	18,401,385
7	50	626	1,849,118	23,046,544	1,503,502	18,738,950
8	24	653	882,249	24,013,413	696,456	18,956,410
9	0	677	0	24,895,662	0	19,080,452

Total	1,815	4,951	66,788,206	182,168,416	62,953,983	155,782,017

* Compared to the original situation, *i.e.*, year−1.

**Table A3.** Avoidable and unavoidable liver cirrhosis cases and costs for men, expected remaining life years.

**Year**	**Cases**	**Cases avoided***	**Unavoidable cost, undisc.**	**Avoidable cost, undisc.**	**Unavoidable cost, disc.**	**Avoidable cost, disc.**
−1	677	0	24,895,662	0	24,895,662	0

0	563	114	20,708,534	4,187,128	20,708,534	4,187,128
1	447	230	16,430,169	8,465,493	15,951,620	8,218,925
2	360	317	13,244,896	11,650,766	12,484,585	10,981,964
3	300	377	11,036,705	13,858,957	10,100,149	12,682,909
4	257	420	9,451,401	15,444,261	8,397,448	13,722,026
5	224	453	8,225,541	16,670,121	7,095,424	14,379,793
6	196	481	7,208,986	17,686,676	6,037,412	14,812,313
7	172	505	6,323,891	18,571,771	5,141,902	15,100,549
8	150	526	5,530,568	19,365,094	4,365,881	15,286,984
9	131	546	4,808,045	20,087,617	3,684,966	15,395,486
10	113	564	4,144,359	20,751,303	3,083,792	15,440,918
11	96	581	3,531,935	21,363,728	2,551,545	15,433,611
12	81	596	2,965,415	21,930,247	2,079,882	15,381,434
13	66	610	2,440,635	22,455,027	1,661,954	15,290,781
14	53	623	1,954,126	22,941,536	1,291,908	15,167,058
15	41	636	1,502,871	23,392,791	964,636	15,014,942
16	29	647	1,084,174	23,811,488	675,621	14,838,532
17	19	658	695,589	24,200,073	420,843	14,641,442
18	9	667	334,882	24,560,780	196,708	14,426,870
19	0	677	0	24,895,662	0	14,197,648

Total	3,305	10,226	121,622,724	376,290,521	106,894,811	274,601,315

* Compared to the original situation, *i.e.*, year−1.
